# Welcome to *Biology of Mood & Anxiety Disorders*

**DOI:** 10.1186/2045-5380-1-1

**Published:** 2011-09-27

**Authors:** Ahmad R Hariri, Lisa M Shin

**Affiliations:** 1 Laboratory of NeuroGenetics, Department of Psychology & Neuroscience, Institute for Genome Sciences & Policy, Duke University, Durham, NC USA; 2 Department of Psychology, Tufts University, Medford, MA USA; 3 Department of Psychiatry, Massachusetts General Hospital and Harvard Medical School, Boston, MA USA

## 

We are witnessing dramatic and exciting shifts in the very foundations of mental health and psychiatry [1-3]. At this time perhaps more than any other it is imperative that research into the biological underpinnings of psychopathology be given a proper outlet to inform, educate and advance these processes in the service of improving the human condition. Indeed, specialized outlets have been created for biological research in a number of disorders including autism, schizophrenia and bipolar disorder [4-6]. Surprisingly, no such dedicated outlet has been available for mood and anxiety disorders, the most common forms of psychopathology, until now.

*Biology of Mood & Anxiety Disorders *(*BMAD*) is a new open access peer-reviewed journal that publishes highly innovative basic, translational and clinical research that advances our understanding of mood and anxiety disorders. The journal welcomes research in all areas relevant to the disorders at the level of their underlying mechanisms. Research areas of interest include but are not limited to: pathophysiology, predictive risk markers, treatment predictors, individual differences and developmental trajectories of mood and anxiety disorders. Experimental approaches include but are not limited to: neuroimaging, psychophysiology, behavioral and molecular genetics, pharmacology, gene-environment interactions, and non-human animal models.

Our idea behind *BMAD *is to create a venue for cutting-edge research that will become the destination of choice for the best work in the field. We are dedicated to providing an outlet for contributions that advance the field in any significant way. We believe that *BMAD *will be an important platform for presenting the findings of those interested in the biology of mood and anxiety disorders at any and all levels. The enthusiastic response from colleagues in neuroscience, psychology, psychiatry, psychophysiology, pharmacology and genetics who have graciously committed their time and expertise by joining the Editorial Board of *BMAD *[7] is testament to the value of this novel venue.

Continued progress in understanding the biology of mood and anxiety disorders is critically dependent on the integration of knowledge gained across many disciplines, methodologies and model systems; currently such knowledge is divided across publications in many journals, none of which is focused entirely on the biology of mood and anxiety disorders. The Editors of *BMAD *thus believe that there is a need for a dedicated journal in this highly diverse field, and given the rising popularity of open access publication, we thought that a new open access journal in our field was particularly timely.

The three publications in this inaugural issue highlight the diversity and breadth of relevant research we seek to encourage and promote at *BMAD*. Fisher and colleagues report on a multimodal PET/fMRI study demonstrating that the balance between excitatory 5-HT_2A _and inhibitory 5-HT_1A _receptors in the medial prefrontal cortex determines, at least in part, the functional responsiveness of the amygdala. These data provide unique insight into specific molecular mechanisms through which a common treatment target, namely 5-HT signaling, modulates a corticolimbic circuitry heavily implicated in the pathophysiology of mood and anxiety disorders. Murphy and Frodl present findings of altered white matter pathways in depression derived from a meta-analysis of diffusion tensor imaging studies. Their meta-analysis reveals specific decreases in the functional integrity of the superior longitudinal fasciculus in patients with major depressive disorder. Finally, Waters and McCormick describe the challenges involved in examining the effect of exogenous corticosterone on anxiety-like and depressive behavior in adolescent rats. Such diversity will continue to be featured in the forthcoming months, including reviews on facial emotion processing in major depressive disorder, evoked potential studies in post traumatic stress disorder (PTSD), and validation criteria in animal models of anxiety and depression, as well as original research on the involvement of the habenula in PTSD.

Open access will allow key findings in our field like those above to be readily accessible and far-reaching because they will appear online and without charge to readers for access. An online journal also means more rapid reviews and shorter time to publication. Articles will be published online immediately upon acceptance and soon after listed in PubMed. For an interdisciplinary field, dispensing new knowledge quickly is critical. A fixed article-processing charge will be levied to cover the publication costs; there are no additional page or color figure charges [8]. We would like to note that many universities are now defraying the costs of open access publishing [9,10] furthering the accessibility of *BMAD *to our community.

While the open access format will allow us to publish articles without the constraint of page limitations, we are pleased to provide a specific forum for Brief Reports of less than 1500 words, which are no longer supported by many journals in psychiatry and neuroscience. We hope that the availability of the Brief Report format will allow for even more rapid publication of highly novel and influential research. Finally, the online format permits the journal to generate a cover for every article we publish. We therefore provide the opportunity for authors of each article to highlight their work through their chosen cover image.

The Editors of *BMAD *are committed to making this new venture a success for all investigators exploring the biological processes underlying mood and anxiety disorders. We hope that you share our vision and will join us in making the journal the primary outlet for research in our field and an invaluable resource not only for our scientific community but for all who share an interest in mental health.
